# Multiple Immunotherapy-Related Cardiovascular Sequelae

**DOI:** 10.1016/j.cjco.2022.11.018

**Published:** 2022-11-29

**Authors:** Sherin M. Menachery, David Chuquin, Ariel Sindel, Andrew Poklepovic, Antonio Abbate, Wendy Bottinor

**Affiliations:** a Department of Internal Medicine, Virginia Commonwealth University Health System, Richmond, Virginia, USA; b Department of Cardiology, Virginia Commonwealth University Health System, Richmond, Virginia, USA; c Department of Hematology-Oncology, Virginia Commonwealth University Health System, Richmond, Virginia, USA

**Use of immune checkpoint inhibitors (ICIs) is a revolutionary therapy, but it is associated with immune-related adverse events (irAEs). An elderly woman with xanthogranuloma disseminatum (XD) presented with constitutional symptoms and an elevated troponin level after receiving pembrolizumab, an ICI associated with relatively rare cardiovascular adverse events. The patient was determined to have acute coronary syndrome, as well as new-onset atrial fibrillation and thyrotoxicosis. We propose that all 3 conditions represent concurrent irAEs related to use of pembrolizumab. This case stresses the importance of considering ICI-related toxicity for patients with multiple events.**

A 71-year-old woman with metastatic XD affecting primarily the face and upper chest was started on pembrolizumab (an anti-PD1 ICI) at a dose of 200 mg intravenous on day 1 of a 21-day cycle. Previous therapies included a 4-month course of cyclophosphamide followed by a 4-month course of trametinib.

An echocardiogram performed 4 months after the initiation of trametinib showed a newly decreased left ventricular ejection fraction (LVEF) of 30%-35%, with global hypokinesis but no focal regional wall-motion abnormalities. Despite multiple risk factors for coronary disease—including type 2 diabetes mellitus, peripheral artery disease with stenting of left external iliac artery and bypass of right femoral artery 3 years prior, hypertension, hyperlipidemia, and tobacco use—myocardial perfusion imaging did not show any quantifiable myocardial perfusion defects. Trametinib was discontinued due to the published association with heart failure. Her medications included aspirin, carvedilol, sacubitril-valsartan, simvastatin, glimepiride, linagliptin-metformin, and hydroxychloroquine.

After 2 cycles of pembrolizumab she presented with severe frontal headache, photophobia, back pain, a fever to 38.2°C, and inability to tolerate oral intake. She later endorsed mild intermittent midsternal chest pain that did not significantly worsen with exertion.

Her electrocardiogram showed normal sinus rhythm with nonspecific horizontal ST depressions less than 1 millimeter in the infero-lateral leads. She subsequently developed new-onset atrial fibrillation with rapid ventricular response that spontaneously converted to normal sinus rhythm. Her labs were significant for the following: initial troponin I level elevated at 1.19 ng/mL (normal level: < 0.06 ng/mL), which then declined on serial measurements; thyroid-stimulating hormone level of 0.01 ulU/mL (normal range: 0.35-4.94 ulU/mL); elevated free T4 level of 2.4 ng/dL (normal range: 0.7-1.5 ng/dL); and elevated total T3 level of 238 ng/dL (normal range: 35-193 ng/dL). No evidence of thyroid receptor antibodies was present. Thyroid ultrasound showed heterogenous echogenicity.

Due to concern for myocarditis, a potentially fatal and more well-known irAE, the decision was made to obtain cardiac magnetic resonance imaging (CMR). CMR revealed an LVEF of 41%, with new hypokinesis in the inferior and lateral walls and a subtle focus of subendocardial delayed enhancement in the basal inferior and inferolateral walls. These findings were more consistent with acute coronary syndrome (ACS) than with myocarditis.

The patient underwent coronary angiography demonstrating a mid-vessel 80% stenosis of the left circumflex artery (Fig. 1), which prompted treatment with a drug-eluting stent and a P2Y-12 inhibitor, as well as medical therapy for coronary artery disease. She was also started on direct oral anticoagulation for atrial fibrillation, and steroids for thyrotoxicosis. A prolonged steroid taper was prescribed, as ICI therapy-mediated irAEs could be a common link for her multiple diagnoses. Pembrolizumab was discontinued, and the patient was trialed on cobimetinib but suffered from disease progression and was switched to binimetinib. CMR performed 8 months after her hospitalization showed an LVEF of 48%, with improved wall motion in the inferior and lateral segments.

**Figure 1:**
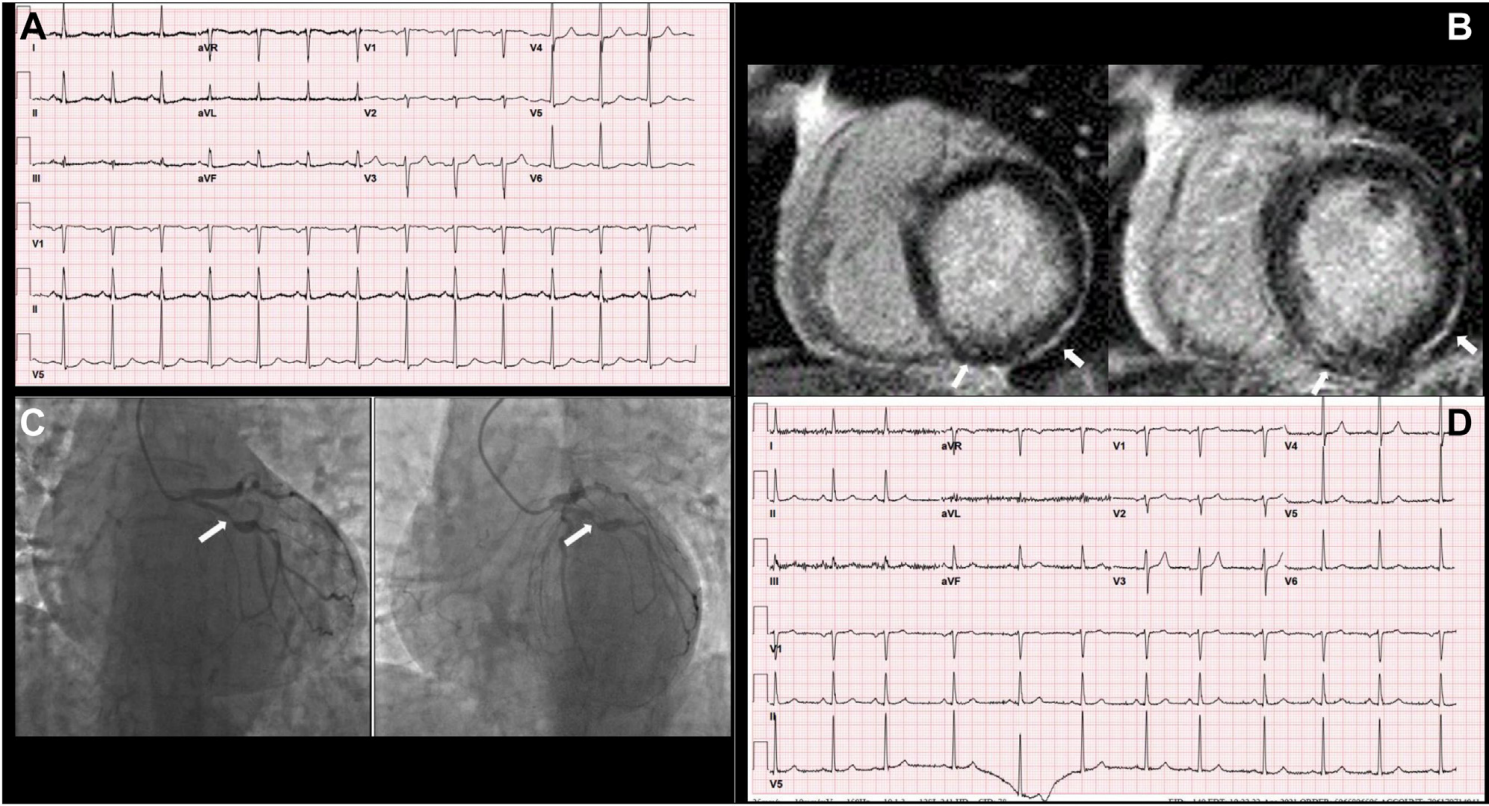
(**A**) Electrocardiogram prior to coronary intervention demonstrates sinus rhythm with nonspecific ST segment changes in the infero-lateral leads. (**B**) Cardiac magnetic resonance imaging demonstrating subtle subendocardial enhancement in the basal inferior and inferolateral walls after gadolinium administration (**arrows**). (**C**) Coronary angiography in the right anterior oblique caudal projection and spider projection demonstrating a large-caliber left circumflex artery, with 80% stenosis of the mid-vessel (**arrow**) followed by poststenotic dilation and subsequent 60%-70% stenosis. (**D**) Electrocardiogram after percutaneous coronary intervention demonstrates improvement in ST segments.

## Discussion

ICIs target inhibitory T-cell signaling, thereby activating the immune system against malignant cells. A consequence of these therapies can be irAEs, which may impact multiple organ systems or cause multiple manifestations in a single organ system concurrently. VigiBase, the global database of the World Health Organization, has identified reports of myocarditis, pericardial disease, arrhythmias, vasculitis, and acute myocardial infarction among individuals receiving ICI therapy, including pembrolizumab (Table 1).^1^ Although these side effects are acknowledged, few cases of multiple concurrent irAEs have been reported.

**Table 1:** Cardiovascular immune-related adverse events (irAEs) from use of immune checkpoint inhibitors

Cardiac irAEs	Pembrolizumab	Atezolizumab	Nivolumab	Cemiplimab	Avelumab	Durvalumab
Arrhythmia						
Tachycardia	386	178	644	14	31	53
Bradycardia	101	22	144	3	11	10
Miscellaneous	150	34	195	12	9	14
Myocarditis	456	87	704	24	29	62
Pericardial disease	269	81	367	4	10	37
Heart failure	278	98	424	16	14	34
Acute coronary syndrome	187	77	322	10	10	28
Cardiac arrest	123	43	171	3	17	25
Structural cardiac disorders	126	28	134	3	7	16
Total adverse cardiac events	1722	561	2742	76	115	245
Total adverse events	40,805	12,415	62,650	1355	2700	5610

Listed are the most frequently reported adverse cardiac events submitted to VigiAccess (World Health Organization, http://www.vigiaccess.org/ [accessed November 5, 2022]) related to use of various immune checkpoint inhibitors. Miscellaneous arrhythmias include bundle branch blocks, sinus node dysfunction, and pulseless electrical activity. Structural cardiac disorders include cardiomyopathy (excluding ischemic), valvular disease, chamber enlargement, and intracardiac mass.

Research has recently elucidated the role of ICI therapy in atherosclerosis-mediated cardiovascular events. At the molecular level, increased uptake in the aorta and major branches, consistent with atherosclerotic inflammation, has been noted on positron emission tomography/computed tomography after an average of 4 months of treatment with ICI, compared to uptake before treatment.^2^ Additionally, patients treated with ICIs had a 3-fold increase in the rate of total plaque volume progression and a 4-fold increase in adverse cardiovascular events in the 2 years following ICI initiation.^3^ Such accelerated atherosclerosis greatly contributed to the 3-fold increased risk for myocardial infarction, coronary revascularization, and ischemic stroke found among individuals receiving ICI therapy, compared with the risk among matched peers.^3^

Several clinical trials further support the role of inflammation in cardiovascular disease. The **C**anakinumab **An**ti-inflammatory **T**hrombosis **O**utcomes **S**tudy (CANTOS) demonstrated that canakinumab, a monoclonal antibody against interleukin-1β, showed a statistically significant dose-dependent reduction in nonfatal myocardial infarction, nonfatal stroke, and cardiovascular death, without affecting cholesterol levels.^4^ Colchicine, an inhibitor of tubulin polymerization that can disrupt neutrophil and cytokine proliferation, also has been studied as a potential therapy for coronary artery disease. In the **Lo**w-**Do**se **Co**lchicine (LoDoCo) trial, patients with stable coronary disease who were randomized to receive colchicine in addition to standard therapy had a significantly lower incidence of acute coronary syndrome, out-of-hospital cardiac arrest, or noncardioembolic ischemic stroke, compared to the incidence among those who received standard therapy alone over a 3-year period.^5^ Although canakinumab and colchicine target proximal components of the inflammatory cascade, other downstream targets of atherosclerotic plaques include T cells.

An immune-modulated process may also explain other ICI-mediated effects, such as atrial fibrillation and thyroid dysfunction. Postmortem tissue analysis has revealed lymphocytic infiltrates within the cardiac conduction system in 2 patients who developed fulminant myocarditis with complete heart block after ICI treatment.^6^ A prospective clinical trial in melanoma patients revealed increased diffuse uptake by the thyroid on positron emission tomography/computed tomography after recent pembrolizumab infusion; these patients developed thyrotoxicosis following inflammatory thyroiditis related to ICI.^7^ Ultimately, widespread activation of lymphocytes in response to ICIs appears to be a shared inciting factor responsible for multi-system autoimmune adverse events. Given the temporality of our patient’s presentation in relation to ICI initiation, the occurrence of multiple events in a short time period, and improvement with irAE-specific treatment, we conclude that pembrolizumab-mediated inflammation affecting multiple organs represents a probable cause for her presentation.

This case emphasizes the need to consider ICI-related toxicity as a unifying diagnosis among patients who present with concurrent irAEs. Moreover, this case demonstrates the utility of advanced imaging in distinguishing among irAEs and providing disease-appropriate therapy in a timely manner. In this patient’s case, CMR was able to rule out myocarditis, a potentially fatal disease if it is not identified early, and allowed for percutaneous coronary intervention for non-ST segment myocardial infarction.

**Novel Teaching Points**

• IrAEs from use of ICIs can arise concurrently.
• Basic science and clinical research reveal a link between immunotherapy-mediated inflammation and cardiovascular disease.
• Advanced cardiac imaging may help clinicians distinguish among different cardiac irAEs and direct disease-appropriate management.
